# Advances in mesenchymal stem cell exosomes: a review

**DOI:** 10.1186/s13287-021-02138-7

**Published:** 2021-01-19

**Authors:** Yaya Tang, Yan Zhou, Hong-Jun Li

**Affiliations:** 1 Key Laboratory of Vaccine Research and Development for Major Infectious Diseases of Yunnan Province, Institute of Medical Biology, Chinese Academy of Medical Sciences and Peking Union Medical College, Kunming, 650118 People’s Republic of China; 2 Kunming Medical University, Kunming, 650500 People’s Republic of China

**Keywords:** Mesenchymal stem cells, Exosomes, Regenerative medicine, Acquisition methods, Biological characteristics, Biological function, Clinical application

## Abstract

Stem cells can be used for regenerative medicine and as treatments for disease. The application of tissue engineering-related transplantation, stem cells, and local changes in the microenvironment is expected to solve major medical problems. Currently, most studies focus on tissue repair and regeneration, and mesenchymal stem cells (MSCs) are among the most common research topics. MSCs are applicable as seed cells, and they represent one of the current hot topics in regenerative medicine research. However, due to storage limitations and because cell senescence occurs during in vitro expansion, their clinical application is challenging. Exosomes, which are secreted by MSCs through paracrine signalling, not only have the same effects as MSCs, but they also have the advantages of targeted delivery, low immunogenicity, and high repairability. This article reviews the acquisition methods, characteristics, biological functions, and clinical applications of exosomes.

## Introduction

In 1983, exosomes were discovered in sheep reticulocytes [1], but they were initially considered cellular waste. In 2007, scholars found that such nanosized vesicles (exosomes) contain proteins, lipids, and RNA, including mRNA and miRNA. Exosomes can be passed to other cells as signalling molecules that exert biological functions, and because of these properties, they will be used in clinical practice in the future (https://ClinicalTrials.gov/). Exosomes are secreted by a variety of cells and exist in almost all body fluids [2], including the blood, saliva, urine, cerebrospinal fluid, and milk. Exosomes derived from human umbilical cord mesenchymal stem cells (HUC-MSCs) have higher neurolysin activity than exosomes derived from HBMSCs [3]. Compared with canine adipose-derived mesenchymal stem cells, canine bone marrow mesenchymal stem cells release more exosomes [4]. Compared with exosomes derived from human bone marrow mesenchymal stem cells, the yield of exosomes isolated from human amniotic fluid mesenchymal stem cells is higher [5]. Among all the proteins in exosomes that are derived from different types of MSCs, half the proteins are similar [6].

Studies have shown that 60% of the proteins in exosomes derived from HUMSCs and HBMSCs are the same and that these proteins are related to cell growth and antioxidant stress. However, proteins with similar functions have also been found in exosomes derived from AMSCs. Whether they are the same as those in exosomes derived from the umbilical cord and bone marrow remains to be determined [7, 8]. Exosomes derived from HUC-MSCs and HBMSCs can inhibit the growth and apoptosis of tumour cells. Exosomes secreted by HASCs can promote tumour growth but have no effect on U87 MG glioblastoma cells [9] (Table 1).

**Table 1:** Mesenchymal stem cell (MSC)-derived exosomes from different sources

Aspect	MSC-derived	Comparison of results	References
Neurolysin activity	HUMSC-derived exosomes	HUMSC-derived exosomes have higher neurolysin activity	[3]
	HBMSC-derived exosomes		
Exosome production	Canine BMSC-derived exosomes	Canine BMSCs release more exosomes	[4]
	Canine AMSC-derived exosomes		
	HAMSC-derived exosomes	Higher yield of exosomes isolated from human amniotic fluid mesenchymal stem cells	[5]
	HBMSC-derived exosomes		
Exosomal components	HUMSC-derived exosomes	60% of the proteins in exosomes are the same	[7, 8]
	HBMSC-derived exosomes		
Tumour mechanism	HBMSC-derived exosome	HUC-MSCs and HBMSCs derived exosomes can inhibit the growth and apoptosis of tumour cells	[9]
	HUMSC-derived exosomes		
	HAMSC-derived exosomes	The exosomes secreted by HASCs can promote tumour growth	

Mesenchymal stem cells (MSCs) have the potential for self-renewal and multidirectional differentiation [10, 11]. Some examples of common MSCs include adipose MSCs (AMSCs), bone marrow MSCs (BMSCs), umbilical cord MSCs (UC-MSCs), and gingival MSCs [12]. MSCs have attracted substantial attention since their discovery in 1968 and have been used in preclinical research for many years [13]. Exosomes are extracellular vesicles with nanoscale bilayer membranes that can be secreted by almost all cell types. Due to the phospholipid bilayer on their surface, exosomes have good stability and permeability. After they enter their target cells, exosomes can regulate the function and signalling of those cells [14].

Exosomes have a cup-shaped structure under the microscope and contain lipids, proteins, and nucleic acids, such as lncRNAs and miRNAs. Exosomes, which range from 30 to 120 nm in diameter [2], are approximately 1.13–1.19 g/ml in density [15]. Exosomes are stored long term at − 80℃. Compared with freshly isolated exosomes, exosomes stored in different conditions over a long period of time will exhibit different degrees of diameter enlargement, and different degrees of protein leakage from exosomes in the supernatant are observed [16–18].

Various types of mesenchymal stem cells can secrete exosomes under normal and pathological conditions, such as in cancer and viral infections. Exosomes are mainly derived from multivesicular bodies (MVBs), which are formed by the invagination of intracellular lysosomal particles, and are released into the extracellular matrix through paracrine signalling after fusion with the endosomal membrane and the cell membrane through endocytosis. MSC-derived exosomes from different sources have similar synthetic pathways [1].

**Fig. 1:**
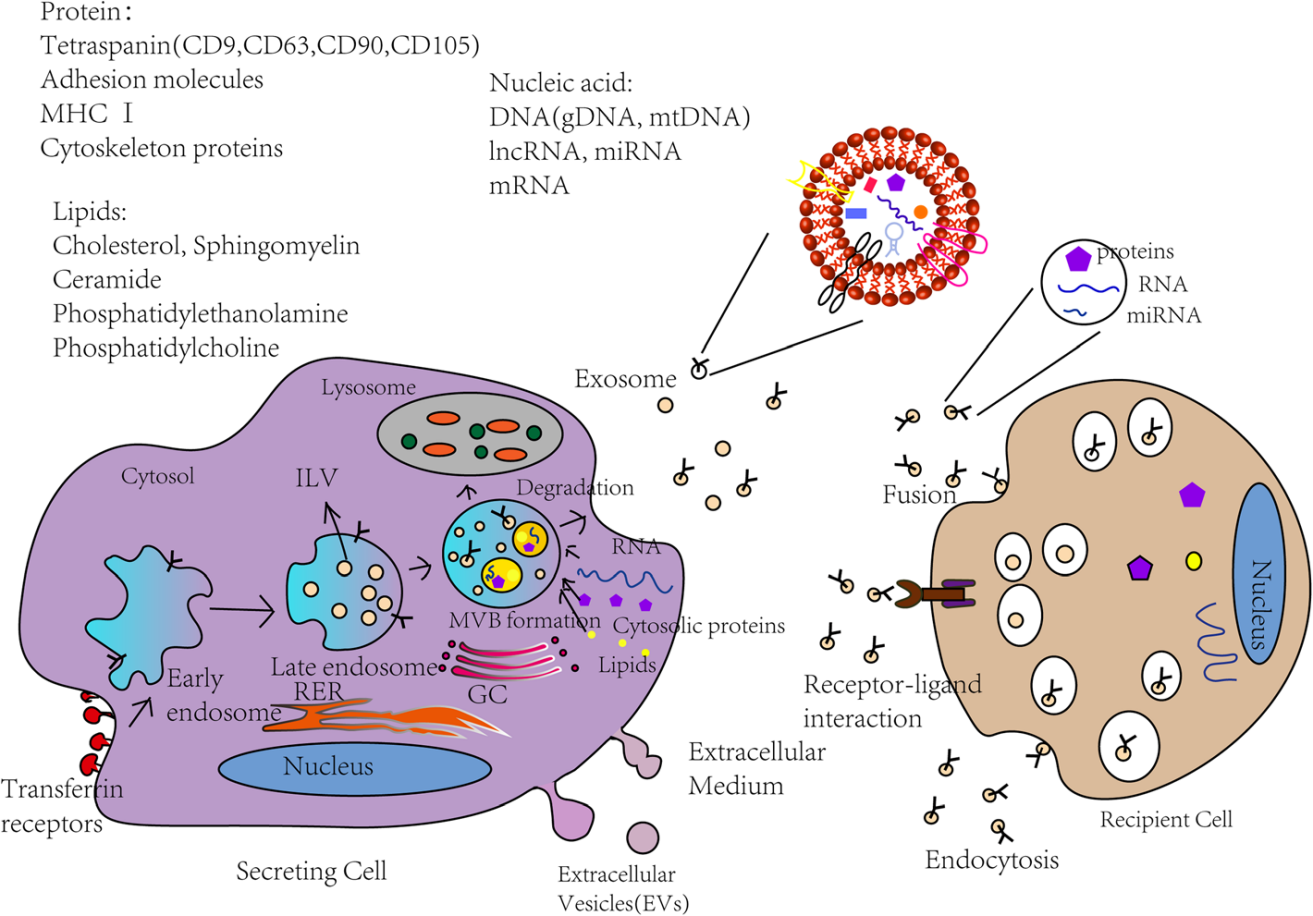
Exosome biogenesis and secretion

Exosomes are produced in very dynamic endosomal organelles in the form of intraluminal vesicles (Fig. 1). First, these organelles undergo different maturation processes to form intraluminal vesicles (ILVs), which are the precursors of exosomes and MVBs [19]. MVBs can fuse with the lysosomal membrane, which causes them to release their contents (including exosomes) into the extracellular matrix [20]. Two secretory mechanisms have been described. The first is the mechanism of sorting complexes from endosomes. The endosomal sorting complex produced by the combination of ILVs and MVBs helps the specific components enter ILVs and form the precursors of exosomes [21]. The other is a complex nonendosomal separation mechanism. This mechanism can assist in the formation of ILVs and MVBs through the auxiliary action of lipids, proteins [22], and other molecules.

## Exosome isolation methods

The following seven methods are currently available for the effective isolation of exosomes: differential centrifugation, precipitation, flushing separation, ultrafiltration, antibody affinity capture, microfluidic separation, and mass spectrometry (MS). These seven methods are compared below (Table 2, Table 3).

**Table 2:** Comparison of the seven methods

Isolation methods	Separation basis	Purity	Recovery rate
Differential centrifugation with addition of 30% sucrose buffer solution	Density, size, etc.	High	Low [23, 24]
Precipitation	Polymer precipitation	Low	High [25–29]
Flushing separation	Positive charge adsorption	High	Low [30]
Ultrafiltration	Size	High	Low [31–34]
Immunomagnetic bead method	Antigen-antibody specific reaction	High	[11, 35–44]
Microfluidic separation	Density, size, etc.	High	[42, 43]
Mass spectrometry	Ion-to-mass ratio	High	[44, 45]

**Table 3:** Comparison of the seven methods

Isolation methods	Advantages	Disadvantages
Differential centrifugation with addition of 30% sucrose buffer solution	High yield Slightly lower contamination	Time-consuming Large amount of sample needed [23, 24]
Precipitation	High separation	Low purification rate Long cycle Susceptible to contamination [25–29]
Flushing separation	Saves time and cost	wash off some exosomes [30]
Ultrafiltration	No chemical reagent contamination Stable structure	Mixed with other particles of similar size [31–34]
Immunomagnetic bead method	High specificity easy operation	Easily affected by pH and salt concentration [11, 35–41]
Microfluidic separation	Small sample requirements, efficient and simple	Lack of standardised clinical samples and large-scale experiments; Small single separation [42, 43]
Mass spectrometry	Good reproducibility, low sample consumption, stable test results	High cost [44, 45]

### Differential centrifugation

The centrifugation speed is either increased in a stepwise manner or lower speeds and higher speeds are alternately applied so that particles with different sedimentation rates are separated in batches at different separation speeds and different centrifugation times. This method is called differential centrifugation. Differential centrifugation is the gold standard for exosome isolation. The latest evidence has shown that the three-time ultracentrifugation method is not only time-consuming and laborious but also fails to achieve good purity. Therefore, scholars have proposed a double ultracentrifugation scheme that involves the addition of 30% sucrose buffer solution during the first centrifugation step. This method has a high yield and slightly lower contamination levels of lipoprotein and AGO2. Therefore, this is a more effective isolation method [23]. The degree of weight balance of exosomes during pregnancy affects term and preterm birth and reflects physiological changes in the ectoderm and can therefore be used as a biomarker and indicator of cell function [24].

### Precipitation

In this process, a solvent is added to the solution to change the polarity and solubility of some components so that they precipitate out into the solution. This method is called precipitation. Differential centrifugation is time-consuming and requires a large amount of sample, but it is the most widely used method. The precipitation method can effectively improve the separation of biological fluids [25]. Methods include isolation using the Serum™, the Exo-Q and Exo-Spin™ blood cell purification kits [16], the mi-RCURY Exosome Separation Kit [26], the Exo Quick-TC Exosome™ Precipitation Solution Kit (System Biosciences, USA) [27], methanol precipitation [28], and the Total Exosome Isolation kit [29].

### Flushing separation

In this case, the sample is added to one end of a chromatographic column, and the mobile phase that is adsorbed or dissolving on the stationary phase is weaker than the components in the sample. This is used as the flushing agent due to the adsorption and dissolution of the components in the sample on the stationary phase. Chromatography separates components in a sample from one another because of the different abilities of the rinsing agent. This method is called flushing separation and provides time- and cost-effective advantages, which makes it a promising method for the clinical application of exosome separation [30].

### Ultrafiltration

Due to the pressure difference between the two sides of an ultrafiltration membrane, which serves as the driving force, this membrane is used as the filter medium. Under a certain pressure, when the original liquid flows over the membrane surface, only water and small molecules can pass through to become the permeate, and the volume is larger than that of the membrane surface. The material with pores is intercepted and becomes a concentrated solution to achieve the purification, separation, and concentration of the original solution. This method is termed ultrafiltration.

Although density gradients can be used to remove impurities such as nonspecific AGO protein [31], as mentioned earlier, ultracentrifugation can be troublesome for large numbers of samples. Moreover, it may affect the separation of protein and RNA from exosomes [32]. Therefore, ultrafiltration should be combined with centrifugal washing and OPTIPREP™ gradient separation, but the column has limitations in that it cannot be reused [33]. After ultrafiltration, the mRNA in the exosomes can be removed [34].

### Immunomagnetic bead method

In this process, the antigen is solidified on the substrate, and the supernatant of the antibody-containing culture medium is added to wash away the unbound antibody. The antibody bound to the antigen can be labelled with fluorescein. This method is called the immunomagnetic bead method. Shed microvesicles can then be continuously immunopurified with anti-A33- and EPCAM-coupled magnetic beads [35]. Single-domain antibodies (VHHs, nanobodies) are structurally stable, easy to design, and capable of labelling specific residues. They are also cheap to produce on a large scale and are clonally stable [36]. Another advantage is that they can directly attack soluble antigens and cells [37–39]. Wang Jing et al. [11] used CD81-labelled immunomagnetic beads to sort AMSC exosomes. Greening et al. used immunoaffinity capture to isolate exosomes from cells of colorectal cancer patients [40]. Researchers have also used specific markers of exosomes to detect the effects of exosome isolation.

Experimental results have shown that exosome isolation by the antibody affinity capture method is more effective than that by the centrifugation method or the density gradient method [41]. An enzyme-linked immunosorbent assay (ELISA) involves the combination of soluble antigen (Ag) or antibody (Ab) to a solid-phase carrier, such as polystyrene. ELISA is a qualitative and quantitative immune reaction detection method that depends on the specific binding of antigen and antibody.

### Microfluidic separation

Microfluidics is a science and technology that is used to precisely control and manipulate fluids on a microscale level. Its main feature is the ability to manipulate fluids in micro/nanoscale spaces. Microfluidics also has basic functions in biological and chemical laboratories, such as in sample preparation. The basic feature and greatest advantage of the ability to scale reactions, separations, and detections to a chip a few square centimetres in size are the flexible combination and large-scale integration of multiple unit technologies on an overall controllable tiny platform. Microfluidics is an interdisciplinary method that involves engineering, physics, chemistry, micro-processing, and bioengineering. This method is called microfluid separation.

Microfluidic technology uses the physical and biochemical properties of dimensional dense bodies for microscale separation, detection, and analysis. In addition to utilising well-established separation methods/influencing factors of separation, this technology uses new sorting mechanisms, such as acoustic, electrophoretic, and electromagnetic operations. The technology is fast and requires only a small amount of sample and few reagents [41]. This technology is not only used to isolate exosomes, but it can also be used in the diagnosis, detection, and differentiation of cancers [42]. Tumour-related exosomes can be detected in the plasma using a microfluidic chip designed with a self-assembled three-dimensional herringbone nanopattern [43].

### Mass spectrometry

The components in the test sample are ionised to generate ions with different charge-to-mass ratios. The ion beam is formed under the action of the accelerating electric field and enters the mass return analyser. The electric field and magnetic field are used to generate opposite velocity dispersion-ion beams. After passing through the electric field, the slower ions will deflect more, while the faster ones will deflect less. In the magnetic field, the ions will be deflected with the opposite angular velocity vector; that is, the slower ions will deflect more and the faster ones will deflect less when the two fields are deflected.

When the effects compensate for each other, their orbits intersect at one point. At the same time, mass separation can occur in the magnetic field so that ions with the same mass-to-charge ratio but with different speeds are focused on the same point. Ions with different mass-to-charge ratios are focused on different points, and they are focused separately to obtain a mass spectrum to determine quality. This method is called separation by mass spectrometry.

Chromatography is used for separation, and MS is used for qualitative detection, and the two are usually performed in combination. Although MS preserves protein profiles, no standard has been established for MS. During experiments, researchers should focus on reducing the risk of introducing experimental artefacts. The use of commercial ED foetal bovine serum (FBS) to remove FBS in exosomes is the first choice for the detection of exosomes using MS because mass spectrometry does not introduce many interfering proteins during detection and provides a good environment for detection. If the correct sample processing method is used, the quality of the MS data is high and can be increased [44]. A method to separate serum exosomes based on QEV column separation is fast and reliable [45].

## Biological characteristics

**Fig. 2:**
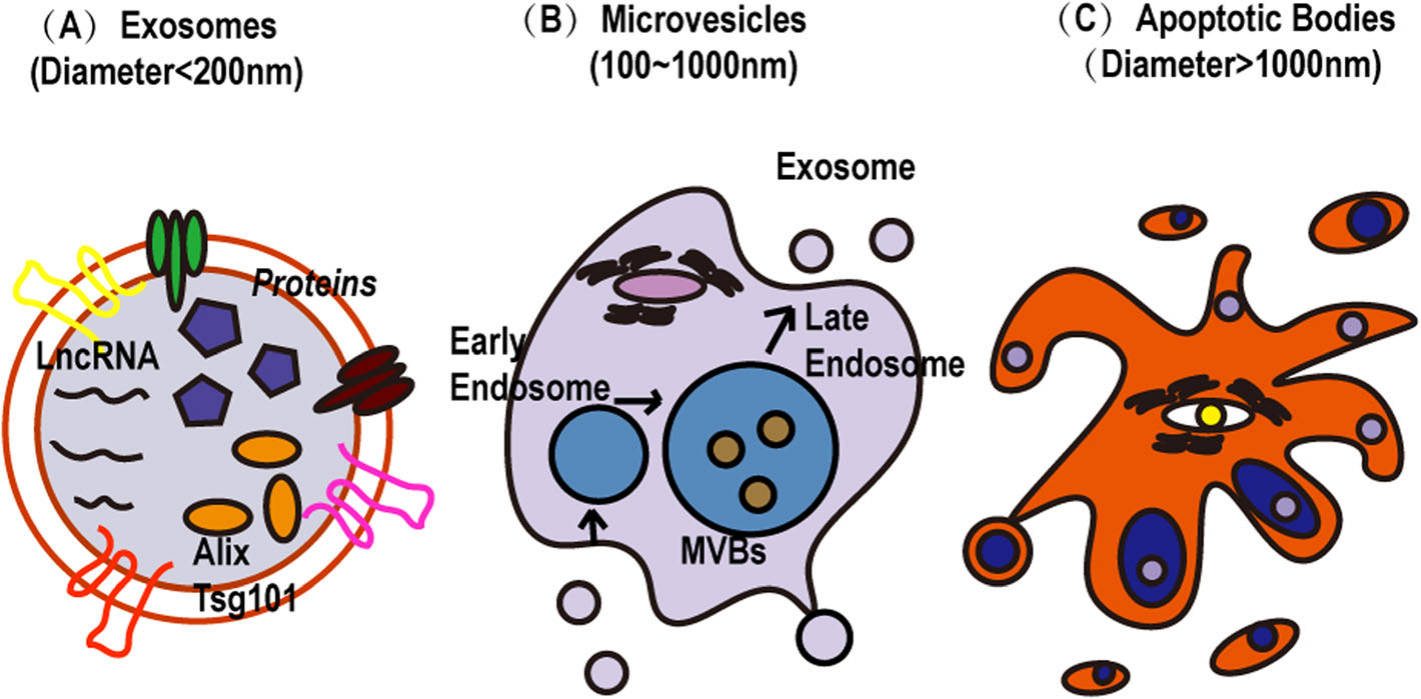
Three types of extracellular vesicles

Extracellular vesicles can be divided into three types: exosomes [46] (diameter < 200 nm), microvesicles (100–1000 nm), and apoptotic bodies (diameter > 1000 nm) (Fig. 2). Exosomes are a subset of extracellular vesicles that are generated by the extracellular secretion of MVBs [1]. To clearly understand the interaction between MSC exosomes, it is necessary to evaluate the physical and chemical characteristics of exosomes, such as their shape, size, surface charge, and density. Several techniques are often used to characterise exosomes, including dynamic light scattering (DLS), transmission electron microscopy (TEM), atomic force microscopy (AFM), tunable resistance pulse sensing (TRPS), nanoparticle tracking analysis (NTA), flow cytometry (FCM), and confocal microscopy [41].

### ASC-derived exosomes

The human body is rich in adipose tissue, and thus, compared with UC-MSCs and BMSCs, AMSCs are abundant with extensive sources and have a high purification yield. Moreover, AMSC removal is painless and inflicts only small trauma, and AMSCs can be obtained from the Plastic Surgery Department. AMSCs can also be used in medical aesthetics. However, AMSCs have strict requirements for storage conditions and have low immunogenicity, which has restricted their clinical application. The membrane structure of exosomes is relatively stable, and AMSC-derived exosomes can be stored for long periods; hence, AMSC-derived exosomes have generated increased interest among researchers [47].

#### Morphological characteristics of ASC-derived exosomes

The primary cell morphology of the adipose tissue is not fixed, and primary cells can be either fusiform or circular. Primary cells can adhere to other cells within 1–2 days of cell culture. Around the fifth passage, they form a single layer and grow in a vortex or radial pattern. Their morphology changes into a single long spindle. Exosomes, which have a uniform round cup shape and a diameter of 30–120 nm, can be observed by scanning electron microscopy. These features distinguish them from apoptotic bodies and microvesicles. The culture of primary AMSCs involves enzymatic digestion and tissue block adherence [48].

#### Composition of ASC-derived exosomes

Exosomes contain lipids, proteins, and RNA. Since the diameter of exosomes is relatively small, they cannot be accurately detected by ordinary flow cytometry. Therefore, for the identification of exosomes by differential centrifugation, flow cytometry, immunomagnetic bead method, and transmission electron microscopy, the surface proteins on exosomes have the same direction as those on the plasma membrane. Membrane-bound proteins include different clusters of differentiation proteins including CD9, CD10, CD13, CD29, CD44, CD63, CD73, CD90, CD105, enkephalin enzyme, and major histocompatibility complex MHC I molecules, among others. The haematopoietic molecular markers CD3, CD19, and CD45 have not been detected on exosomes. CD44 is similar to CD105 and is expressed in almost all human MSC types. CD90 is highly expressed in various MSCs [11, 49].

In addition, AMSCs express a marker similar to CD81, which is similar to what is expressed in BMSCs. However, no single-specific surface marker of AMSCs has been identified thus far [50]. Exosomes can be characterised with antibody chips that detect certain marker proteins, including annexins (such as FLOT1), ICAM1, EPCAM, fat valve structural proteins (such as ANXA5), and the multivesicular biosynthesis-related proteins ALIX and TSG101. Exosomes do not contain GM130. The cellular origin and formation process of exosomes affect their protein content [47, 50–52].

The RNA in exosomes mainly includes mRNA and noncoding RNA. Noncoding RNAs include miRNAs and long noncoding RNAs (lncRNAs). LncRNAs play roles in signal transmission and in the regulation of various diseases [50]. Exosomal lipids include cholesterol, sphingomyelin, ceramide, and phosphatidylserine, and of these, ceramide plays a key role in the formation of exosomes [53–56].

### HUC-MSC-derived exosomes

The umbilical cord, which connects the mother and the foetus and supplies foetal nutrition during pregnancy, is of placental origin. Human MSCs can be divided into four types: HUC-MSCs, human umbilical cord perivascular MSCs (HUCPV-MSCs), human umbilical cord Wharton’s Jelly MSCs (HWJ-MSCs), and human amniotic membrane-derived MSCs (HA-MSCs) [57].

#### Morphological characteristics of HUC-MSC-derived exosomes

Primary UC-MSCs are spindle-shaped, and no obvious vortex growth is observed. After 1 day of direct adherent culture by the primary cell tissue mass method, a small number of cells adhere and grow, and the cells show a vortex growth pattern after the second generation. Some cells exhibit a vortex growth pattern during passage to the fifth generation at which point the cells become long and fusiform with the typical characteristics of vortex growth. Exosomes vary in size, but have a diameter of approximately 30–100 nm [2, 58]. They appear as round or elliptical membranous vesicles that can be aggregated and distributed and have clear boundaries around the membranous structure [59].

#### Composition of HUC-MSC-derived exosomes

Flow cytometric analysis of HUC-MSCs showed that HUC-MSCs are positive for the cell-specific surface markers CD29, CD44, C/73 (SH3), CD90 (Thy-1), and CD105 (SH2) and negative for CD11b, CD34, and CD45. Exosomes secreted by UC-MSCs express the exosome-specific four-transmembrane protein markers CD9, CD63, and CD81 and the multivesicular biosynthesis-related protein ALIX, as analysed by Western blot. The protein levels of the four transmembrane proteins could be used as biomarkers. Notably, different techniques used to isolate exosomes result in the detection of different expression levels of these proteins. In addition, these exosomes contain lipids and miRNAs, such as miR-26b and miR-206 [57, 59–65].

### BMSC-derived exosomes

These exosomes can interact with many types of cells and are not easily inactivated. BMSCs can be isolated from the bone marrow. BMSCs have the advantages of a low infection rate of pathogenic microorganisms, stable biological performance, low immune rejection after transplantation, and a high number of possible passages [66].

#### Morphological characteristics of BMSC-derived exosomes

After 1–2 days of adherent culture under hypoxia and other conditions, some cells become adherent, and the size and shape of the cells are different. The morphology of adherent cells is mostly round. When the cells are cultured for 4–5 days, the number of adherent cells gradually increases, and the cells begin to colonise. Their morphology is still diverse, and cells appear polygonal and fusiform. After the second passage, the morphology changes to that of a single fusiform shape, and the cells tend to grow radially towards the vortex. After passaging to the fourth generation, the MSCs grow in a vortex pattern. These exosomes are uniform in size, 30–100 nm in diameter, and cup-shaped, with a clear model structure around them [67].

#### Components of BMSC-derived exosomes

Exosomes secreted by paracrine BMSCs were analysed by Western blot. The exosomes contained cystine, the four-transmembrane proteins CD9, CD63, CD81, HSP70, syntenin-1, and multivesicular biosynthesis-related protein TSG101. Osteoclast-derived exosomes also contain receptor activator of NF-κB (RANK) [66, 67].

Most RNAs in exosomes are pre-microRNAs and include miR-301, miR-let-7a, and miR-22. BMSC surface markers include the antigen integrin family member CD29; the adhesion molecules CD44, CD73, CD90, CD11b, CD105, and CD106; and the Ly-6 antigen family members SCA-1 and Grn94. Grn94 is found only in BMSCs and not in exosomes. BMSC-derived exosomes are negative for CD14, CD34, and CD45 [66–69].

## Biological functions

The function of MSCs depends on their abilities to interact with recipient cells and to transfer their protein, lipid, and RNA content to these cells. MSCs are widely used in tissue repair and in acute and chronic injuries. Exosomes contain specific cell binding sites and can thus transport substances into target cells [70]. The biological functions of exosomes derived from three types of mesenchymal stem cells were summarised (Table 4). No substantial differences were observed in exosomes from these cell types, and these exosomes share similar functions.

**Table 4:** The biological characteristics of exosomes from three sources

HAD-MSC-derived exosomes	HUC-MSC-derived exosomes	HBMSC-derived exosomes
Tissue repair and regeneration [71] Angiogenesis [71] Inflammation regulation [71] Transplantation rejection [71] Osteogenesis and adipogenesis spread [71] Healing of the skin [11, 71] Reduction in apoptosis [74] Immune system regulation [74]	Inflammatory response [77] Ossification [78] Promote new blood vessel formation [79] Pathological damage regulation [80] Nerve regeneration [80]	Inhibit the secretion of interferon γ by lymphocytes [69, 81, 82] Immunomodulatory [83] Stimulate tissue repair [83] Pro-migration [83] Stimulate proliferation [83] Anti-inflammatory and antioxidant effects [83] Regulate osteoclast generation [83] Osteogenic differentiation [83]

### HASC-derived exosomes

These exosomes are secreted by paracrine cells and play a key role in tissue repair and regeneration. Not only do they promote angiogenesis, but they also upregulate the early inflammatory response to improve transplant rejection reactions and induce osteogenesis and adipogenesis. Cell proliferation [11, 71] will accelerate healing of the skin but will also promote the migration and invasiveness of melanomas [72, 73]. Exosomes can also inhibit apoptosis and regulate the immune system [74].

### HUC-MSC-derived exosomes

HUC-MSCs have stronger in vitro expansion and multidirectional differentiation capabilities than umbilical cord tissue-derived MSCs [75, 76]. Exosomes derived from umbilical cord mesenchymal stem cells can inhibit the secretion of IL-6 by macrophages by knocking down the large-conductance Ca2+ -activated K+ (BKCa) channel to enhance the secretion of exosomes by WJ-MSCs, which then leads to immunoregulation of the inflammatory response [77]. The combination of HUC-MSC-derived exosomes and hydrogels can promote the ossification of mouse osteogenic progenitor cells (mOPCs) [78].

When exosomes isolated from HUC-MSCs are cocultured with human cutaneous vein endothelial cells in vitro, the tubular structure of endothelial cells is more obvious, and endothelial cells demonstrate better migratory properties. HUC-MSC exosomes can promote new blood vessel growth [79]. Olfactory ensheathing cells are beneficial for nerve regeneration, but they are limited by the hypoxic environment. HUC-MSC-derived exosomes can regulate the pathological damage caused by hypoxia. The combination of both olfactory ensheathing cells and HUC-MSC-derived exosomes also promotes nerve regeneration [80].

### HBMSC-derived exosomes

Exosomes secreted by BMSCs can inhibit the secretion of interferon γ by peripheral blood mononuclear cells, such as lymphocytes [69, 81, 82] and have been shown to exhibit immunomodulatory, tissue repair stimulatory, promigratory, proliferative, anti-inflammatory, and antioxidant effects; they have also been shown to contain regulatory proteins. The miRNAs in these exosomes can regulate osteoclast generation, osteogenic proliferation and differentiation of BMSCs [83].

## Clinical application

MSCs can upregulate regulatory T cells (Tregs) and downregulate Th17 cell development through paracrine-secreted exosomes, which participate in immune balance regulation. They can be used as “cell-free” biological therapies, which have opened up new research directions for autoimmune diseases [60]. The clinical application of exosomes derived from three types of mesenchymal stem cells was summarised (Table 5).

**Table 5:** The clinical application of exosomes from three sources

HAD-MSC-derived exosomes	HUC-MSC-derived exosomes	HBMSC-derived exosomes
Enhance the formation of blood vessels in fat transplantation [70] Promote wound healing [71] Regulate albinism and pigmentation in Moynahan syndrome [71] Improve multiple scleroderma [70] Improve the prognosis of melanoma, and reduce hair loss [71] Increase the sensitivity of liver cancer to chemotherapy drugs [84] Promote apoptosis of tumour cells and inhibit prostate cancer cell proliferation [84] Improve Dox-induced cardiomyopathy [85] Protect the cartilage matrix in osteoarthritis [86] Improve motor and sensory functions [87]	Inhibition of transplanted vein intimal hyperplasia [88] Inhibition of endometrial cancer cell proliferation; tumour homing abilities [89] Reduce neurological damage and brain oedema; neuroprotection, anti-ageing effect, used in human aesthetic treatments [90] Inhibit infection with hepatitis C virus (HCV) [88, 89] Development of COVID-19 drugs [91, 92]	Promotion of myeloma cell proliferation [1, 84] Inhibition of multiple myeloma cell proliferation [20] Immunomodulators; form vascular structures and support haematopoiesis [93] Improve osteoporosis and treatment of cartilage damage, osteoarthritis, and Alzheimer’s disease (AD) [93] Transmit signals and proteins [94] Antagonise the cellular effects of U87 MG glioblastoma cells [15]

### HAMSC-derived exosomes

Adipose stem cell (ASC)-derived exosomes can enhance the formation of the blood vessels in fat transplantation [71]. Although they can promote wound healing, they can also lead to the migration and invasiveness of melanomas through the fatty acid oxidation pathway. Exosomes have immunomodulatory functions and can regulate microglia polarisation. They can also improve inflammation. Exosomes can regulate albinism and pigmentation in Moynahan syndrome and can also be used in the treatment of diseases, such as multiple sclerodermas; in addition, they can improve the prognosis of melanoma and reduce hair loss [71]. Exosomal miRNAs can be detected in the cell microenvironment and can also be transferred between cells. These miRNAs can inhibit tumours and can also act as cell regulators under various physiological and pathological conditions. The miRNAs and proteins of these exosomes can be used as noninvasive prognostic biomarkers and as in vivo markers of disease detection. Exosomes increase the sensitivity of liver cancer to chemotherapy drugs by transferring miR-122 into cells. MiR-145, which is transported by exosomes, promotes apoptosis of tumour cells and inhibits the proliferation of prostate cancer cells by reducing caspase 3 and 7 pathway activity [84].

It has been reported that exosomes secreted by MSCs treated with macrophage migration inhibitors can protect the heart with the same long noncoding RNA/microRNA [85]. Exosomes can also be used as potential therapeutic targets for the treatment of Dox-induced cardiomyopathy [86] and can downregulate the inflammatory response and reduce oxidative stress, which can protect the cartilage matrix in osteoarthritis. The treatment of AMSCs using different methods, such as hypoxia, can promote exosome secretion. Exosomes can effectively treat spinal cord injury by inhibiting the expression of inflammatory factors, which effectively protects motor and sensory functions [87].

### HUC-MSC-derived exosomes

HUC-MSC-derived exosomes are more frequently used in the treatment of various diseases than exosomes secreted by other types of stem cells. Simultaneously, exosomes can accelerate endothelialisation, thereby strengthening the endothelium, inhibit transplanted vein intimal hyperplasia, and promote cell proliferation and migration [88]. MiR-302A in these exosomes can inhibit the proliferation of endometrial cancer cells. In addition, these exosomes have tumour homing abilities [89].

MiR-206 can reduce neurological damage and brain oedema caused by the BDNF/TRKB/CREB signalling pathway and can inhibit neuronal apoptosis, preventing early brain injury and improving neuroprotection, and when combined with acupuncture in mice, miR-206 has shown a significant anti-ageing effect. If developed well, these exosomes may be used in human aesthetic treatments [90]. HUC-MSC-derived exosomes can inhibit viral replication and inhibit hepatitis C virus (HCV) infection, but the specific mechanisms are not yet defined. One explanation is that a certain RNA or protein may act on a specific target, which can then interfere with the proliferation of the hepatitis C virus. This is because the hepatitis C virus does not easily spread and will not ultimately develop into cirrhosis. These exosomes can also downregulate the “cytokine storm” to restore oxygenation. Finally, these exosomes can be used to develop COVID-19 drugs [91, 92].

### HBMSC-derived exosomes

The pre-microRNAs found in these exosomes are all associated with immune-related pathways [83]. Exosomes can be beneficial to tumour growth, proliferation, and migration [1, 84]. Gastric cancer cells are more sensitive to exosomes than osteosarcoma cells, and exosomes can be used to treat patients with gastric cancer. BMSC-derived exosomes promote the proliferation of myeloma cells in patients with multiple myeloma. However, some research has shown that exosomes inhibit tumour activity, and exosomes secreted by the normal human bone marrow have been shown to inhibit the proliferation of multiple myeloma cells [20].

MiR-22 and miR-let-7a are used clinically as immunomodulators, as they are beneficial to the formation of vascular structures, exert neuroprotective effects, and provide haematopoietic support. Exosomes can effectively improve osteoporosis and can treat cartilage damage, osteoarthritis, and Alzheimer’s disease (AD) [93].

Katsuda et al. reported that MSCs can produce exosomes with different contents, which act as signal transmitters and protein transporters between target cells and MSCs [94]. Lopez-Verrilli et al. showed that exosomes from different sources had significantly different effects on the growth of synapses in primary cortical neurons and dorsal root ganglion explants [3]. Additionally, HASCs, HUC-MSCs, and HBMSCs can antagonise the cellular effects of U87 MG glioblastoma cells [9].

## Summary

Basic research on exosomes and the exploration of clinical treatments have received extensive attention. Exosomes can be derived from different types of MSCs, and exosomes from different sources have unique characteristics. On the one hand, they share the characteristics of tissue repair, regeneration, and promotion of angiogenesis. On the other hand, AMSC exosomes can be used for wound healing, while HUC-MSC-derived exosomes have tumour homing ability and can also inhibit viral infection and replication. Recent studies have found that exosomes are also effective in the treatment of COVID-19.

According to the results of studies in which exosomes have been used to treat certain diseases, the mechanisms of applying exosomes to different diseases are also different. In tumour research, exosomes have a dual regulatory role. HUC-MSC- and HBMSC-derived exosomes can inhibit the proliferation of glioblastomas and promote apoptosis. However, exosomes secreted by HASCs can promote tumour growth but have no effect on the apoptosis of U87 MG glioblastoma cells [9]. The reason for these completely opposite results of treatment with exosomes from different sources is still unclear.

In terms of neurodegenerative diseases, one of the causes of Alzheimer’s disease is a mutation that causes increased β-amyloid, and neurolysin is a β-amyloid peptide-degrading enzyme. Studies have found that HUMSC-derived exosomes have higher neurolysin activity than HBMSC-derived exosomes. This is the only difference in the function of these exosomes from different sources, which is worthy of further in-depth study. This may provide new ideas for the treatment of Alzheimer’s disease [3].

In a study of intervertebral disc degeneration, Xia et al. found 150 proteins that are closely related to the enhancement of the inflammatory response [95]. Exosomes can significantly inhibit the inflammatory response of apoptotic nucleus pulposus cells. Proteins in BMSC-derived exosomes can restore function in nucleus pulposus cells with mitochondrial damage, restore normal structure, and reduce the oxidative response of mitochondria. These potential mechanisms mainly serve to promote the anti-inflammatory response, inhibit cell apoptosis, and restore mitochondrial function in the repair of intervertebral disc degeneration, but the detailed mechanism is still unclear.

Studying the mechanism of exosomes in the treatment of diseases is the primary connection to future clinical research. As a new treatment concept, exosomes have special advantages. As a signalling molecule, MSC exosomes not only exert the same effects as MSCs, but they also have a more stable membrane structure than MSCs. Compared with whole-cell therapy, MSC-derived exosomes are well tolerated and have low immunogenicity. These advantages provide broader prospects for disease treatment.

However, according to the data and research results obtained thus far, many unclear links and problems remain. Current problems involve the following: the technology of large-scale culture and isolation of MSCs; the optimal method for the long-term preservation of exosomes; the rapid and accurate determination of the exosome concentration and quality control, purification, and transplantation conditions of exosomes; and the cost and safety of exosomes. Resolution of these issues requires more in-depth analysis for clarification. Although many problems with exosomes still exist, the prospects of basic research and clinical applications are worthy of attention and exploration.

## Supplementary Information

**Additional file 1.** Abbreviations and disclaimer

## Data Availability

Data sharing is not applicable to this article, as no datasets were generated or analysed during the current study.

## Abbreviations

MSCs: Mesenchymal stem cells
AMSCs: Adipose mesenchymal stem cells
BMSCs: Bone marrow mesenchymal stem cells
UC-MSCs: Umbilical cord mesenchymal stem cells
MVBs: Multivesicular bodies
ILVs: Intraluminal vesicles
MS: Mass spectrometry
AGO2: AGO2 protein
ELISA: Enzyme-linked immunosorbent assay
FBS: Foetal bovine serum
DLS: Dynamic light scattering
TEM: Transmission electron microscopy
AFM: Atomic force microscopy
TRPS: Tunable resistance pulse sensing
NTA: Nanoparticle tracking analysis
FCM: Flow cytometry
MHC I: Major histocompatibility complex I
CD: Leukocyte differentiation antigen, cluster of differentiation
FLOT-1: Flotillin-1
ALIX: Programmed cell death 6-interacting protein (PDCD6IP)
TSG101: Tumour susceptibility gene 101 protein
GM130: Golgi matrix protein 130
ICAM-1: Intercellular cell adhesion molecule-1
EpCAM: Epithelial cell adhesion molecule
ANXA5: Annexin A5
miRNA: MicroRNA
lncRNA: Long non-coding RNA
HUC-MSCs: Human umbilical cord mesenchymal stem cells
HUCPV-MSCs: Human umbilical cord perivascular mesenchymal stem cells
HWJ-MSCs: Human umbilical cord Wharton’s Jelly mesenchymal stem cells
HA-MSCs: Human amniotic membrane-derived mesenchymal stem cells
BKCa: Large-conductance Ca2+-activated K+ channel
mOPC: Mouse osteogenic progenitor cell
HSP70: Heat shock protein 70
Tregs: Regulatory T cells
Th17: T helper cell
ASC: Adipose stem cell
HCV: Hepatitis C virus
COVID-19: Novel coronavirus
AD: Alzheimer’s disease
