# Supplementary material for: Advances in mesenchymal stem cell exosomes: a review
Source: Stem Cell Res Ther. 2021 Jan 19;12:71. doi: 10.1186/s13287-021-02138-7 (PMC7814175; doi:10.1186/s13287-021-02138-7)
Supplement: Supplementary file 1 — Additional file 1. Abbreviations and disclaimer [file 13287_2021_2138_MOESM1_ESM.docx]

**Open Access**This article is licensed under a Creative Commons Attribution 4.0 International License, which permits use, sharing, adaptation, distribution and reproduction in any medium or format, as long as you give appropriate credit to the original author(s) and the source, provide a link to the Creative Commons licence, and indicate if changes were made. The images or other third party material in this article are included in the article's Creative Commons licence, unless indicated otherwise in a credit line to the material. If material is not included in the article's Creative Commons licence and your intended use is not permitted by statutory regulation or exceeds the permitted use, you will need to obtain permission directly from the copyright holder. To view a copy of this licence, visit http://creativecommons.org/licenses/by/4.0/. The Creative Commons Public Domain Dedication waiver (http://creativecommons.org/publicdomain/zero/1.0/) applies to the data made available in this article, unless otherwise stated in a credit line to the data.

**Abbreviations**

| MSCs | Mesenchymal stem cells | | | | |  |  |  |
| --- | --- | --- | --- | --- | --- | --- | --- | --- |
| AMSCs | Adipose mesenchymal stem cells | | | | |  |  |  |
| BMSCs | Bone marrow mesenchymal stem cells | | | | |  |  |  |
| UC-MSCs | Umbilical cord mesenchymal stem cells | | | | |  |  |  |
| MVBs | Multivesicular bodies | |  |  |  |  |  |  |
| ILVs | Intraluminal vesicles | | |  |  |  |  |  |
| MS | Mass spectrometry | |  |  |  |  |  |  |
| AGO2 | AGO2 protein | |  |  |  |  |  |  |
| ELISA | Enzyme-linked immunosorbent assay | | |  |  |  |  |  |
| FBS | Foetal bovine serum | | |  |  |  |  |  |
| DLS | Dynamic light scattering | | |  |  |  |  |  |
| TEM | Transmission electron microscopy | | |  |  |  |  |  |
| AFM | Atomic force microscopy | | |  |  |  |  |  |
| TRPS | Tunable resistance pulse sensing | | | |  |  |  |  |
| NTA | Nanoparticle tracking analysis | | |  |  |  |  |  |
| FCM | Flow cytometry | |  |  |  |  |  |  |
| MHC I | Major histocompatibility complex I | | | | | |  |  |
| CD | Leukocyte differentiation antigen, Cluster of differentiation | | | | | |  |  |
| FLOT-1 | Flotillin-1 | | |  |  |  |  |  |
| ALIX | Programmed cell death 6-interacting protein (PDCD6IP) | | | | | | | |
| TSG101 | Tumour susceptibility gene 101 protein | | | | |  |  |  |
| GM130 | Golgi matrix protein 130 | | | | |  |  |  |
| ICAM-1 | Intercellular cell adhesion molecule-1 | | | |  |  |  |  |
| EpCAM | Epithelial cell adhesion molecule | | | |  |  |  |  |
| ANXA5 | Annexin A5 | |  |  |  |  |  |  |
| miRNA | MicroRNA |  |  |  |  |  |  |  |
| lncRNA | Long non-coding RNA | | | |  |  |  |  |
| HUC-MSCs | Human umbilical cord mesenchymal stem cells | | | | | |  |  |
| HUCPV-MSCs | Human umbilical cord perivascular mesenchymal stem cells | | | | | | |  |
| HWJ-MSCs | Human umbilical cord Wharton’s Jelly mesenchymal stem cells | | | | | | | |
| HA-MSCs | Human amniotic membrane-derived mesenchymal stem cells | | | | | |  |  |
| BKCa | Large-conductance Ca2+-activated K+ channel | | | | | |  |  |
| mOPC | Mouse osteogenic progenitor cell | | | | | |  |  |
| HSP70 | Heat shock protein 70 | | | |  |  |  |  |
| Tregs | Regulatory T cells | | | |  |  |  |  |
| Th17 | T helper cell 17 | | | |  |  |  |  |
| ASC | Adipose stem cell | | |  |  |  |  |  |
| HCV | Hepatitis C virus | | |  |  |  |  |  |
| COVID-19 | Novel coronavirus | |  |  |  |  |  |  |
| AD | Alzheimer’s disease | |  |  |  |  |  |  |

**Disclaimer**

Content

PMC is a repository of content primarily from two sources: peer reviewed journals that overall have met NLM’s standards for PMC; and peer reviewed author manuscripts deposited in compliance with the public access policy of NIH or other collaborating funders.

PMC is not a publisher and does not publish journal articles itself. Once a journal as a whole has been accepted for inclusion in PMC, NLM does not judge the quality of individual articles and relies on the scientific publishing process to identify and address problems through published comments, corrections, and retractions. NIH and other funders do not dictate the journals in which their funded authors may publish. Consequently, author manuscripts in PMC may be from journals that have not yet undergone scientific review by NLM, are traditionally out of scope for the NLM collection, or have not met NLM’s standards for PMC.

In addition, as of June 2020 PMC includes preprints reporting NIH supported research in support of the NIH Preprint Pilot. As preprints are interim research products that have not been peer reviewed, readers should be aware that any aspect of the research, including the results and conclusions, may change as a result of peer review.

The presence of an article in PMC does not reflect an endorsement of, or concurrence with, the contents of the article by NLM.

If you have concerns about a specific article (e.g., possible plagiarism, point of view expressed), NLM encourages you to notify the PMC helpdesk, as well as the editors and publisher of the journal.

Liability

For documents and software available from this server, the U.S. Government does not warrant or assume any legal liability or responsibility for the accuracy, completeness, or usefulness of any information, apparatus, product, or process disclosed.

Endorsement

NCBI does not endorse or recommend any commercial products, processes, or services. The views and opinions of authors expressed on NCBI's Web sites do not necessarily state or reflect those of the U.S. Government, and they may not be used for advertising or product endorsement purposes.

External Links

Some NCBI Web pages may provide links to other Internet sites for the convenience of users. NCBI is not responsible for the availability or content of these external sites, nor does NCBI endorse, warrant, or guarantee the products, services, or information described or offered at these other Internet sites. Users cannot assume that the external sites will abide by the same Privacy Policy to which NCBI adheres. It is the responsibility of the user to examine the copyright and licensing restrictions of linked pages and to secure all necessary permissions.

Pop-Up Advertisements

When visiting our Web site, your Web browser may produce pop-up advertisements. These advertisements were most likely produced by other Web sites you visited or by third party software installed on your computer. The NLM does not endorse or recommend products or services for which you may view a pop-up advertisement on your computer screen while visiting our site.

Medical Information and Advice

It is not the intention of NLM to provide specific medical advice but rather to provide users with information to better understand their health and their diagnosed disorders. Specific medical advice will not be provided, and NLM urges you to consult with a qualified physician for diagnosis and for answers to your personal questions.
